# An evaluation of DNA double strand break formation and excreted guanine species post whole body PET/CT procedure

**DOI:** 10.1093/jrr/rrab025

**Published:** 2021-05-24

**Authors:** Tanmoy Mondal, Amit Nautiyal, Somiranjan Ghosh, Christopher A Loffredo, Deepanjan Mitra, Chabita Saha, Subrata Kumar Dey

**Affiliations:** Department of Biotechnology, Maulana Abul Kalam Azad University of Technology, Salt Lake, Kolkata 700064, India; Institute of Nuclear Medicine & Molecular Imaging, AMRI Hospitals, Dhakuria, Kolkata 700029, India; Department of Biology, Howard University, Washington, DC 20059, USA; Department of Oncology, Georgetown University, Washington, DC 20057, USA; Institute of Nuclear Medicine & Molecular Imaging, AMRI Hospitals, Dhakuria, Kolkata 700029, India; Department of Biotechnology, Maulana Abul Kalam Azad University of Technology, Salt Lake, Kolkata 700064, India; Department of Biotechnology, Maulana Abul Kalam Azad University of Technology, Salt Lake, Kolkata 700064, India

**Keywords:** positron emission tomography/computed tomography (PET/CT), ^18^F fluoro-2 deoxy-D-glucose (^18^F -FDG), 8-OHdG, DNA damage, DNA repair, double strand breaks (DSBs)

## Abstract

Ionizing radiation-induced oxidation and formation of deoxyribonucleic acid (DNA) double strand breaks (DSBs) are considered the exemplar of genetic lesions. Guanine bases are most prone to be oxidized when DNA and Ribonucleic acid (RNA) are damaged. The repair processes that are initiated to correct this damage release multiple oxidized guanine species into the urine. Hence, the excretion of guanine species can be related with the total repair process. Our study quantified the total DSBs formation and the amount of guanine species in urine to understand the DNA break and repair process after whole body (WB) exposure to ^18^F-FDG positron emission tomography/computed tomography (PET/CT). A total of 37 human participants were included with control and test groups and the average radiation dose was 27.50 ± 2.91 mSv. γ-H2AX foci assay in the collected blood samples was performed to assess the DSBs, and excreted guanine species in urine were analyzed by a competitive ELISA method. We observed a significant increase of DNA damage that correlated well with the increasing dose (*p*-value 0.009) and body weight (*p*-value 0.05). In the test group, excreted guanine species in urine sample significantly increased (from 24.29 ± 5.82 to 33.66 ± 7.20 mg/mmol creatinine). A minimum (r^2^ = 0.0488) correlation was observed between DSBs formation and excreted guanine species. A significant difference of DNA damage and 8-OHdG formation was seen in the test group compared to controls. Larger population studies are needed to confirm these observations, describe the fine-scale timing of changes in the biomarker levels after exposure, and further clarify any potential risks to patients from PET/CT procedures.

## INTRODUCTION

There is always an increasing demand for oncologic diagnosis and management based on the results of positron emission tomography (PET)/computed tomography (CT) scanning. Hospitals and clinics are also installing more PET/CT scanners because of the versatility in its clinical applications [1]. PET uses a small amount of radioactive material called radiotracer which is detect by the scanner, and the acquired images are superimposed with X-ray-based CT scans for a better understanding of the affected area of the body. As PET/CT is a combination of two different modalities, the effective radiological dose is also a combination of the dose of PET and the dose of CT, resulting in an increased radiation exposure to the patients compared to single modality alone.

**Table 1:** Clinical characteristics of study population

Clinical characteristics	Control Group^*^	Test Group^*^	Combined Group^*^
Age (Years)	40.11 ± 7.14	44.5 ± 10.48	43.43 ± 9.87
Weight (Kg)	65 ± 7.35	63.71 ± 12.43	64.0 ± 11.32
CBG (mg/dl)	106.67 ± 18.48	104.93 ± 19.50	105.35 ± 19.01
Sex (M/F)	5/4	14/14	19/18
Total received dose by test group (mSv)		27.50 ± 2.91	

*Note:*  ^*^Results are displayed are mean ± SEM

**Fig. 1.**
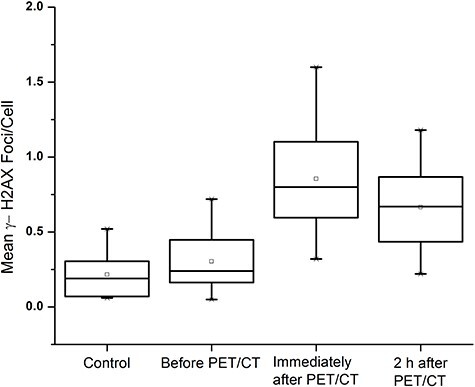
Histogram of meanγ-H2AX foci/cell scored in control and patients samples (before, immediately after and 2 h after PET/CT).

**Fig. 2.**
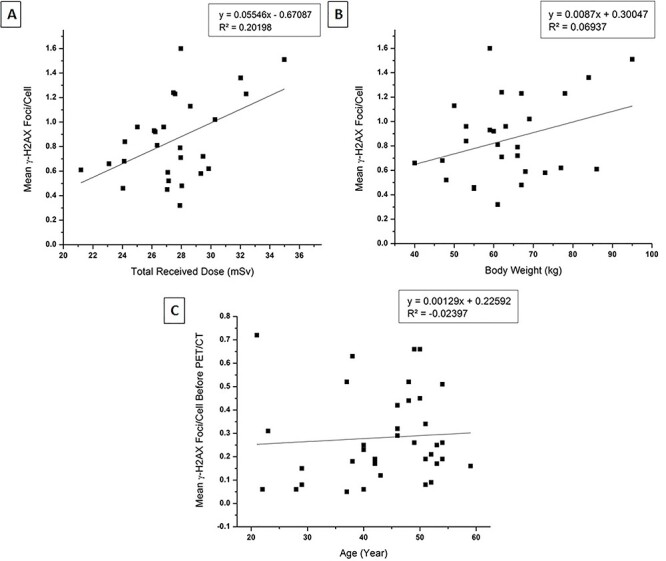
Graphical representation of associated factors responsible for γ-H2AX foci formation. Significant association was noted with total received dose and patients body weight (A and B) *p*-value<0.05. Between age and mean γ-H2AX foci formation was not well significant (C) *p*-value 0.69.

**Fig. 3.**
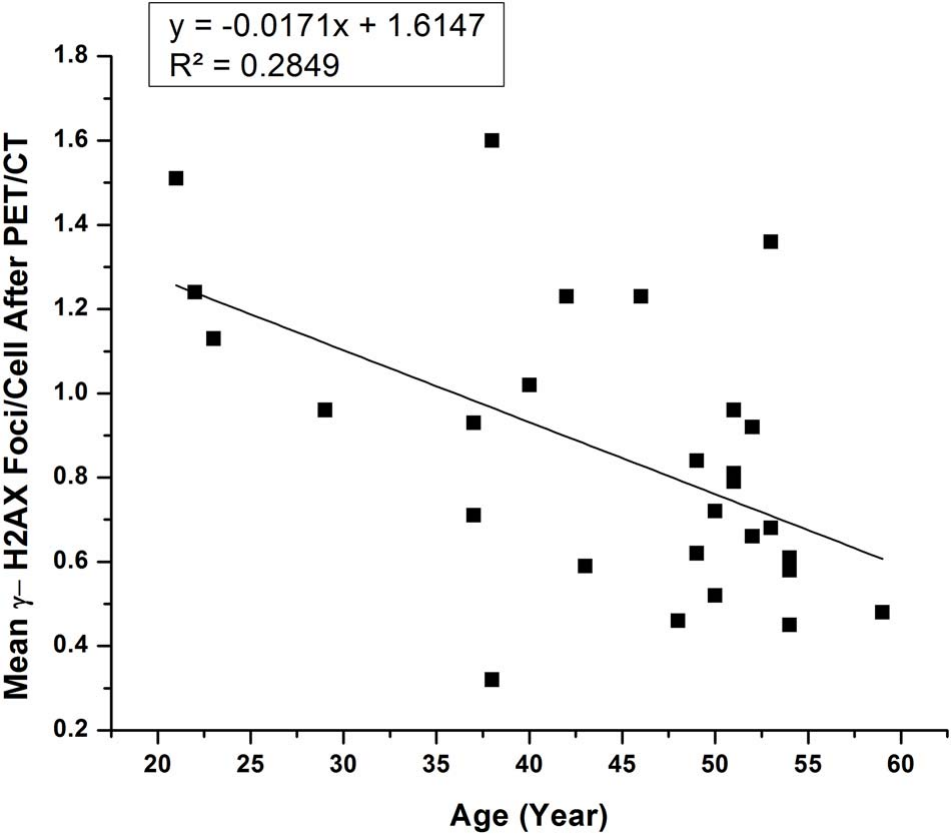
Graphical representation of correlation between γ-H2AX foci formation after PET/CT investigation and age of recruited patients.

**Fig. 4.**
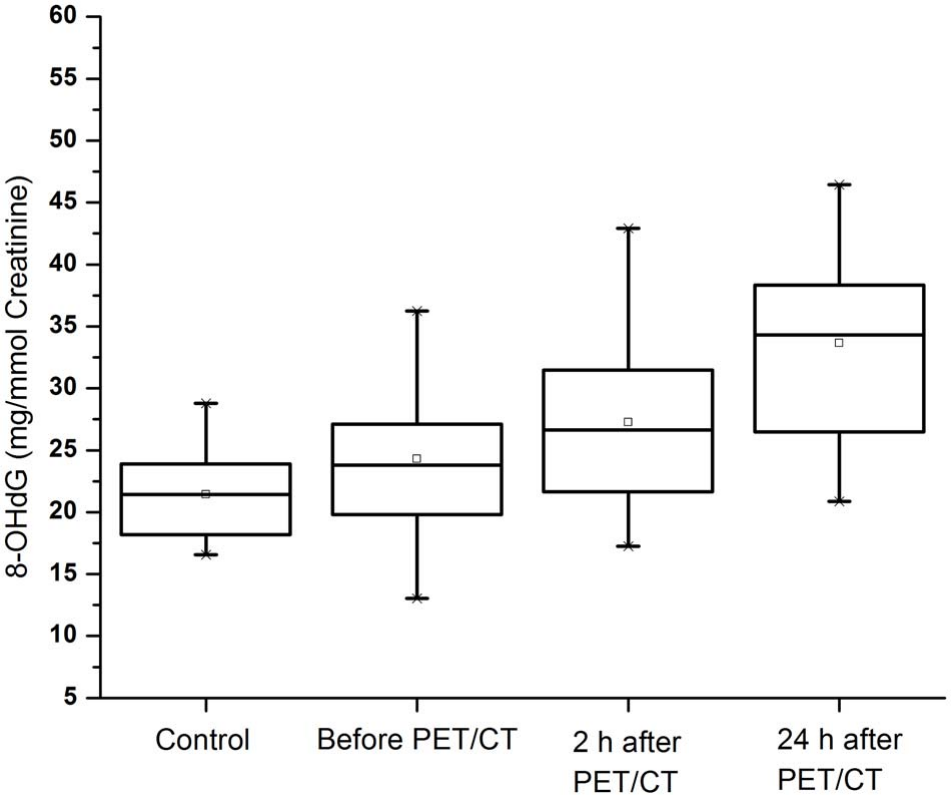
Histogram of quantified guanine species (represented as 8-OHdG) in control and patients samples (before, 2 h after and 24 h after PET/CT).

Currently, the use of whole body (WB) 2-[^18^F] fluoro-2 deoxy-D-glucose (^18^F-FDG)-PET/CT is becoming increasingly popular in the diagnosis of abnormal metabolic activity, and other many types of disease processes [2]. As with any radiation exposure, the medical use of ^18^F-FDG PET/CT raises concerns regarding the possibility of radiation-induced toxicity [3, 4]. In general, PET/CT involves intravenous injection of 10 mCi, which yields 350 MBq of ^18^F-FDG and imaging initiated an hour later. The average yearly natural background radiation dose typically ranges from about 1.5 to 3.5 mSv/year, but a single PET/CT scan strategy can provide an effective dose of 15 mSv, equivalent to 6.4 years of background radiation [5, 6].

Ionizing radiation induced DNA double-strand breaks (DSBs) formation represents the most biologically deleterious type of lesion and results in persistent malfunctioning of cells, and is thought to induce both mutagenesis and carcinogenesis [7, 8]. Reported epidemiological data from exposed human populations demonstrates that doses of 50–100 mSv in protracted exposure or 10–50 mSv acute exposure to ionizing radiation increases the risk of some cancers [9]. These epidemiological data cannot always provide a risk estimate below these levels [10], and are based at least partly on linear extrapolations from existing high-dose data [11–13]. With a linear, no-threshold hypothesis based on mechanistic considerations, low-dose hypersensitivity, delayed genomic instability and induced DNA repair mechanisms are important to measure but difficult to reconcile [14–19]. *In vitro* studies have been helpful in this regard, but human equivalence needs further exploration.

Our previous study observed that ^18^F-FDG induced DSBs formation in isolated peripheral blood lymphocytes and chromosomal aberrations in a V79 cell line [20, 21]. We also observed DNA breaks by comet and micronucleus assay after exposure to PET/CT procedure [22]. In the present study, we extended that work through quantifying the DSBs formation in patients’ blood samples by γ-H2AX assay, and assessed the total DNA RNA oxidative damage burden by quantifying urinary 8-hydroxy-2′-deoxyguanosine (from DNA), 8-hydroxyguanosine (from RNA) and 8-hydroxyguanine (DNA and RNA) levels. As DNA and RNA become oxidized due to radiation exposure, and guanine is most prone to oxidation, the repair processes initiated to correct the damage release multiple oxidized guanine species in urine. Hence by measuring the excretion of guanine species one can also quantify the total repair process. Therefore, we sought to fill the above gaps in knowledge by quantifying total DSBs formation and the amount of guanine species in urine to understand the DNA break and repair process after WB ^18^F-PET/CT.

## MATERIALS AND METHODS

### Study population and recruitment

Participants were recruited at the Institute of Nuclear Medicine & Molecular Imaging, AMRI Hospitals, Dhakuria, Kolkata, between February 2019 and November 2019. The Institutional Ethical Committee approved this study and all patients provided written informed consent. A total of 37 participants were recruited, including those who volunteered for the control group (five male, four female; mean age 40.11 ± 7.14, range 28–46) without PET/CT procedure, and 28 volunteer in the test group (14 male, 14 female; mean age 44.5 ± 10.48, range 22–59) who visited our center for cancer staging and evaluation of pyrexia of unknown origin (PUO) and underwent the PET/CT procedure. The characteristics of the study population are summarized in Supplementary Table S1. The average received dose by the test group was 27.50 ± 2.91 mSv.

### Exclusion criteria

Participants with a history of smoking, diabetes, or cardiopulmonary disease were excluded. Also, volunteers with renal failure, those unwilling to provide written informed consent, those who had received contrast media during any other diagnostic investigation within the week prior to this study, or had received any treatment of chemotherapy/radiotherapy and/or were pregnant were excluded from the study.

### Research participants preparatory (pre-imaging) procedure

Patients were instructed to fast overnight, avoid any kind of physical or stress activity and drink one liter of water 1 h before the examination. An intravenous cannula was inserted in the patient’s vein for an injection of ^18^F-FDG before the examination. All patients were asked to sit in a post-injection waiting room and restrict their physical activity, including talking, to an absolute minimum.

### PET/CT imaging protocol

One hour before the PET/CT procedure 8 MBq/kg ^18^F-FDG was injected intravenously, patients were instructed to empty their bladder in the radioactive toilet before the scan in the GE Discovery 690 PET/CT scanner [23]. Patients were positioned inside the scanner for contrast-enhanced WB PET/CT examination, and were injected with Iohexol, Omnipaque 300 based on the patient body weight at dose of 84.57 ± 16.69 ml and flow rate of 2–4 ml/s (GE Healthcare; Shanghai co., Ltd. Shanghai, China) [24]. A scout scan (in which the patient first undergoes an ‘overview’ or ‘scout’ scan procedure during which X-ray projection data are obtained to identify the axial extent of the CT and PET study), breath-holding lung CT, WB CT and PET (from head to mid-thigh) and low-dose cine CT (provides cross-sectional millisecond tomography that is used for the correction of motion due to diaphragmatic movement) were obtained in an arms-up position and the imaging parameters for WB CT were followed according to our previous study [22]. After completion of CT, the attenuated corrected emission images were acquired for 2 min per bed position in a 3-dimensional mode for a total acquisition time of approximately 15–20 mins.

### Internal dose estimation

Absorbed dose (DT) to a tissue or organ (T) resulting from the intravenous injection of radioactivity (A) of ^18^F-FDG was estimated by using the dose coefficients as recommended by the International Commission on Radiological Protection (ICRP) in Publication 106 (ICRP 2007) for different organs and tissues of the adult MIRD phantom. The following equations were used:

(1) \begin{equation*} {D}_T=A\cdot{\Gamma}_T^{FDG} \end{equation*}

where ${\varGamma}_T^{FDG}$ is dose coefficient recommended by ICRP inpublication 106 for variety of organs and tissue.

The effective dose (E) from ^18^F-FDG WB PET was calculated as follows:

$E=\sum_T{W}_T\cdot{D}_T=A\cdot \sum_T{W}_T\cdot{\varGamma}_T^{FDG}=A\cdot{\varGamma}_E^{FDG}$ (2).

where ${W}_T$ is Tissue weighting factor provided by ICRP Publication 103, and ${\varGamma}_E^{FDG}$ is dose coefficient recommended by ICRP in publication 106 for the WB [25].

### External dose estimation

To determine the radiation dose of patients resulting from CT scans, volume CT dose index (${\mathrm{CTDI}}_{\mathrm{vol}}$) was used. The radiation dose CT component was estimated from dose length product (DLP). DLP was estimated from volumetric ${\mathrm{CTDI}}_{\mathrm{vol}}$ and the scan length for each patient. Values of ${\mathrm{CTDI}}_{\mathrm{vol}}$ and DLP were directly obtained from the display screen of the PET/CT operating console. DLP was calculated from ${\mathrm{CTDI}}_{\mathrm{vol}}$ as:

(3) \begin{equation*} \mathrm{DLP}={\mathrm{CTDI}}_{\mathrm{vol}}\times \mathrm{Scan}\ \mathrm{length}. \end{equation*}

The total effective dose (ED) from the CT scan was estimated using the sex-specific conversion factors k (mSv/mGy cm) [26], where:

(4) \begin{equation*} \mathrm{ED}=\mathrm{DLP}\times \mathrm{Conversion}\ \mathrm{factor}. \end{equation*}

### Blood, urine sample collection and lymphocytes separation

Blood (in 4 ml heparin tube container) and urine (in 30 ml plastic container) samples were collected from the control group at only one single point of time. Blood samples from the test group were collected at three different time points: before PET/CT, immediately after PET/CT and 120 min after the PET/CT examination. Likewise, urine samples were collected at three different points of time: before PET/CT, 120 min after PET/CT and at 24 h later or early morning void. The first voided morning specimen (immediately after waking) is particularly valuable because it provides a time average for biomarker concentrations that may occur during the hours of sleep (approximately eight hours) and also over the course of the day, as the composition and concentration of urine changes continuously [27].

**Fig. 5.**
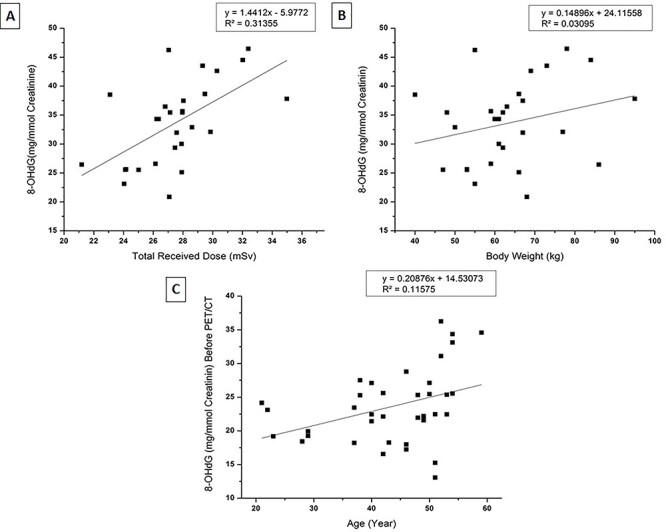
Graphical representation of associated factors responsible for urinary 8-OHdGexcretion. Significant association was noted with total received dose, patients body weight and age (A, B &C) *p*-value<0.05.

**Fig. 6.**
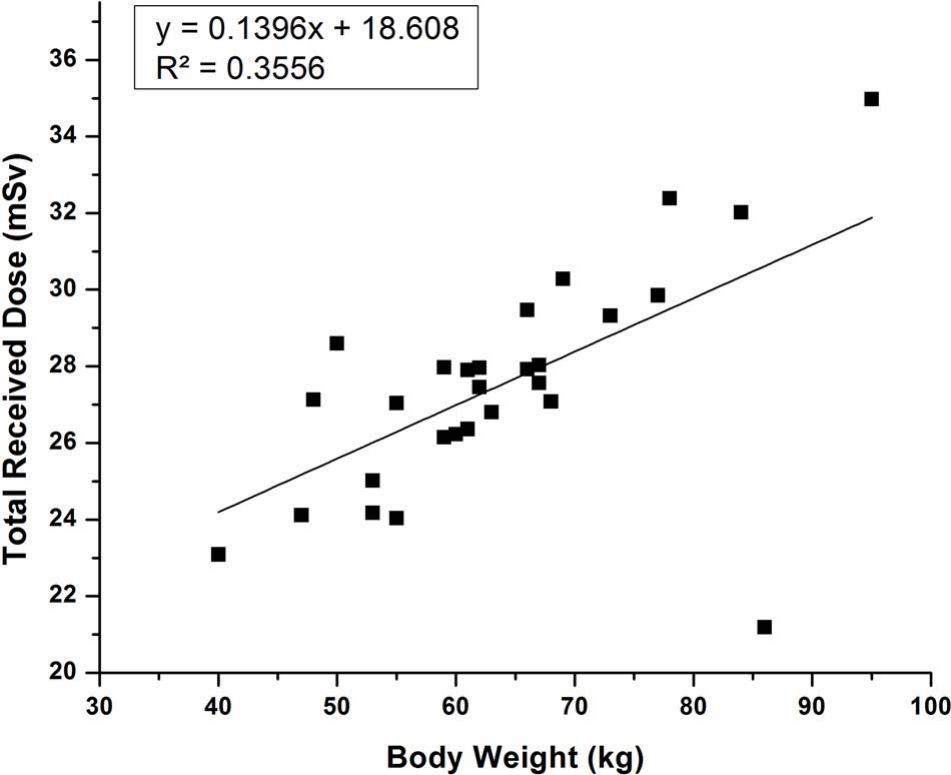
Graphical representation of correlation between total received dose (mSv) and body weight (kg) of the recruited patients.

**Fig. 7.**
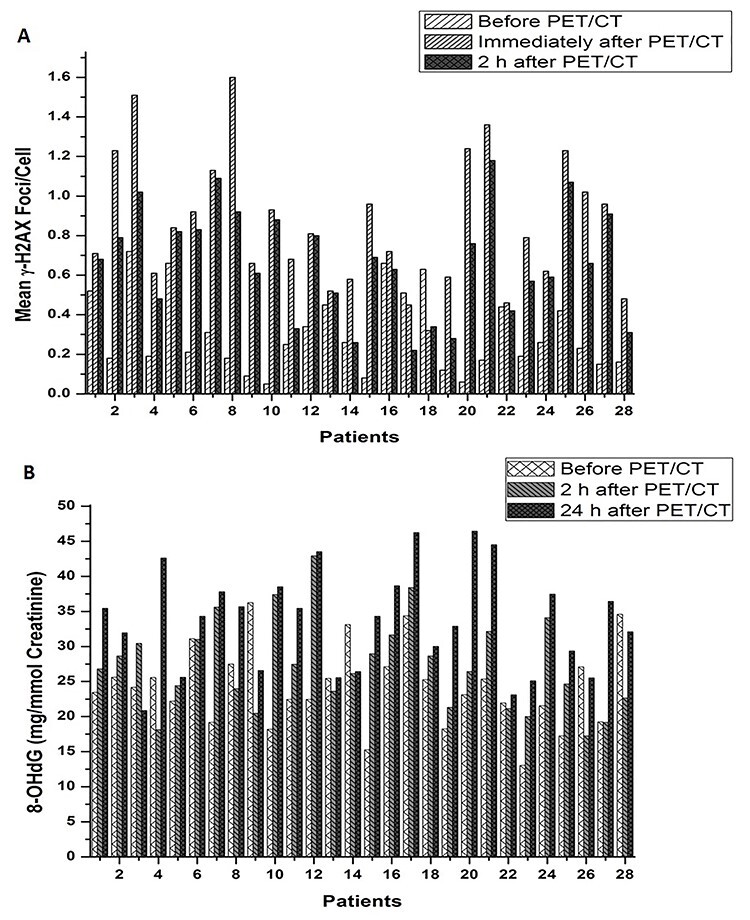
Histogram of inter-individual variation in γ-H2AX foci formation (A) and 8-OHdG excretion (B) at each time change.

**Fig. 8.**
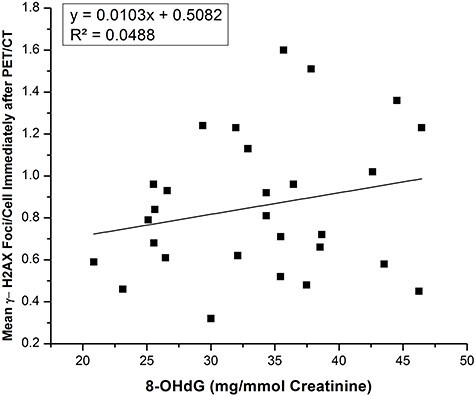
Graphical representation of correlative analysis between mean γ-H2AX foci formation and urinary 8-OHdGexcretion *p*-value 0.25.

### γ-H2AX foci analysis

Briefly, before γ-H2AX foci analysis, all samples were incubated at 37°C, 5% CO_2_ for 30 min to allow phosphorylation of H2AX histones [21]. Isolated lymphocyte cells were fixed in 4% paraformaldehyde (pH 7.4) for 15 min and washed three times in PBS (5 min each). Fixed cells were then permeabilized on ice using 0.1% Triton X-100 (15 min) and again washed gently with PBS; then they were blocked in 10% FBS and 0.1% Tween 20 PBS for 30 min and incubated with mouse anti-H2AX phosphorylated monoclonal antibody (ser 139) clone (Millipore, UK) in the dark for 2 h at room temperature thereafter. After washing slides with 0.1% Tween 20 PBS, cells were incubated with Alexa Fluor 488 goat anti-mouse IgG secondary antibody (1:500) (Invitrogen, Germany) for 1 h at room temperature. Cells were washed four times in PBS for 5 min each time and mounted by using Vectashield mounting medium with 4,6-diamidino- 2-phenylindole. Images were obtained by using fluorescence microscope (Leica DM300, Leica Microsystems, Wetzlar, Germany). At least 100 cells were analyzed for each measurement [20].

### Estimation of excreted guanine species

DNA/RNA oxidative damage was analyzed by the quantification of urinary 8-hydroxy-2′-deoxyguanosine (from DNA), 8-hydroxyguanosine (RNA) and 8-hydroxyguanine (DNA and RNA) levels. The assay was performed by a specialized ELISA kit purchased from Cayman Chemical, USA. The protocol is based on the competitive analysis between oxidatively damaged guanine species and an 8-OHdG-acetylcholinesterase conjugate for a limited amount of DNA/RNA oxidative damage-detecting monoclonal antibody. Because the amount of tracer is held constant while the concentration of oxidatively damage guanine varies, the amount of tracer that can bind to the monoclonal antibody will be inversely proportional to the concentration of oxidatively damaged guanine in the well. The antibody-oxidatively damaged guanine complex binds to the goat polyclonal anti-mouse IgG that has been previously attached to the well. The product of this enzymatic reaction has a distinct yellow color and absorbs strongly at 412 nm. The assay has a detection range from 10.3–3,000 pg/ml and a sensitivity of approximately 30 pg/ml. The level was normalized with creatinine (Cayman Chemical) [28].

### Statistical analysis

Statistical analysis was performed using Student’s t-test for test vs control group comparisons. Reported data are represented as means ± SEM for three independent experiments. Linear regression analysis was performed using OriginPro 8 software. We considered differences statistically significant at *p* < 0.05.

## RESULTS

### Study population characteristics

Table 1 shows the distributions of age, gender and other parameters in the test and control groups. Average body weights in the test and control groups were 63.71 ± 12.43 kg and 65 ± 7.35 kg respectively (*p*-value 0.38), with a combined range of 40 to 95 kg. The mean capillary blood glucose (CBG) in the test group was 104.93 ± 19.50 mg/dl and in the control group was 106.67 ± 18.48 mg/dl (*p*-value 0.40). The mean age in the control group was 40.11 ± 7.14 compared to 44.5 ± 10.48 in the test group (*p*-value 0.12). The age range in the control group was 28 to 40 years, compared 21 to 59 in the test group.

### Quantification of DNA double strand breaks

The mean number of DSB foci in the control group, and for the test group at time points before PET/CT, immediately after PET/CT and after 2 h sample group, were 0.21 ± 0.15, 0.30 ± 0.20, 0.85 ± 0.33 and 0.67 ± 0.27 respectively (Fig. 1). A significant increase of mean γH2AX foci formation observed with the total received dose in the treatment group after immediate sampling (Fig. 2a) and was significantly larger than the control group (*p-*value < 0.05). Inter-individual variation was also observed in γ-H2AX foci formation (Fig. 7a) at each point of time. The results also showed that the mean γH2AX foci formation increased in relation to the body weight of the patients (Fig. 2b), but was inversely related to the patient’s age (Fig. 2c and Fig. 3).

### Estimation of excreted 8-Hydroxydeoxyguanosine quantification

In the nine control samples, the average level of normalized urinary 8-OHdG/creatinine (mg/mmol) was 21.44 ± 3.83 mg/mmol creatinine, whereas in the 28 treated samples the 8-OHdG level was significantly increased to 33.66 ± 7.20 mg/mmol creatinine; in the latter samples there was also a dose–response relationship. Before PET/CT and in the 2 h after PET/CT, the 8-OHdG was 24.29 ± 5.82 and 27.27 ± 6.51 mg/mmol creatinine respectively (Fig. 4). Inter-individual variation was also observed (Fig. 7b). The formation of 8-OHdG increased with the increasing of total received dose during PET/CT procedure (Fig. 5a) and was also increased in association with increasing body weight and age (Figs 5b and c). No significant relationship was observed between γH2AX foci formation and 8-OHdG in the treated samples (Fig. 8 and Supplementary Fig. Fig. S2).

## DISCUSSION

Imaging procedures like CT, single-photon emission CT (SPECT) and PET is highly useful diagnostic modalities in the health sector, but their widespread use has also raised concerns about possible adverse effects of radiation [29]. Our previous studies, both *in vitro* and *in vivo*, have revealed that a significant amount of DSBs is generated after contrast-enhanced PET/CT examination [20, 21, 22]. Another study by Prasad *et al.* [30] reported that conventional ^18^F-FDG-based PET/CT scanning induces DNA damage and chromosomal aberrations in blood lymphocytes. There are no such studies that investigated the influence of ^18^F-FDG PET/CT scanning and DNA DSBs on urinary excretion of 8-OHdG concentration levels. In the present study we quantified the DSB generation by γH2AX method, which is very sensitive biomarker, and quantified the formation of 8-OHdG. The control group samples showed a low but measurable level of foci formation, because DSBs can be caused naturally by a variety of factors like reactive oxygen species (ROS), metabolic processes, deficient repair, programmed biological processes and other causes [31]. The number of γH2AX foci increased markedly in the test group after PET/CT scanning. We also observed that the immediately-collected sample in the test group showed a higher frequency of foci formation than the sample collected 2 h later, possibly reflecting host repair response mechanisms that rapidly reduced the detectable foci formation [32].

Since WB PET/CT is followed by considerable amount of radiation dose, risk–benefit ratios should be carefully weighed prior to every investigation. In our *in vivo* study, the mean γH2AX foci increased proportionally with the increasing total received dose, and the accumulation of even a small amount of mis-repaired DNA damage may be carcinogenic. Previously many studies have also reported the relationships between radiation exposure by PET/CT and formation of γH2AX in lymphocytes [30, 33, 34]. Prasad *et al.* reported that, compared to conventional CT, the ^18^F-FDG PET/CT scanning instrument delivers higher doses to patients, inducing γH2AX foci in blood lymphocytes [30]. Cheezum *et al.* [33] reported the appearance of γH2AX foci in 101 female patients who had undergone coronary CT angiography, and a very recent study by Schumann *et al.* [34] showed that even at very low absorbed doses to the blood of less than 3 mGy, the number of γH2AX in the blood was significantly increased compared to baseline. So, in a general point of view, the dose of up to 32 mSv per study adding to the background radiation is non-negligible [35]. Therefore, PET/CT scanning protocols should be optimized for reducing doses and their associated cancer risks.

In the study protocol, we included some baseline parameters like total received dose, body weight and age to check any associated relationship with enhancement of DNA damage. The observed data clearly shows a direct relationship between total received dose and the number of foci formation. Patients body weight is also one of the important dependable factor with the formation of γH2AX foci, because it is directly proportional to the total received dose. In a general PET/CT procedure the activity of ^18^F prepared for a patient was determined by his or her body weight [36] so that, the total received dose also changes with the administrated activity: this is why we saw a significant (r^2^ = 0.3556) relationship (Fig. 6) between the body weight and total received dose. Interestingly we also observed a trend for increasing formation of H2AX foci with increasing age in the control group, but not in the test group (Supplementary Fig. Fig. S1). Age-related DNA breaks formation is well corroborated by many authors [37, 38].

Several studies have reported an association between increased 8-OHdG and carcinogenesis in various types of malignant tumors [39, 40]. Laboratory measurement of urinary 8-OHdG’s offers some advantages also in this regard, as in addition to being noninvasively measured in urine with high stability, it reflects the overall level of oxidative DNA damage and repair from all cells in the organism [41]. The first voided morning urine sample is very important in response to nullify the other activity which are directly associated in elevation of 8-OHdG. Over the course of the day, the composition and concentration of urine changes continuously. The first voided morning specimen (upon waking) is particularly valuable because it provides a time average for biomarker concentrations that may occur during the hours of sleep (approximately 8 hours) [42]. In contrast, a late morning or afternoon urine sample may reflect dietary, physical and environmental (i.e. tobacco smoke, pollution) exposures. According to Rodrigues *et al*. and Abusoglu *et al*. [28, 43] 8-OHdG levels can be high in some stressful situations including smoking, aging, lack of or extreme exercise and occupational exposure to chemicals. With the increasing of DNA DSBs in patients sample we also observed the elevation of 8-OHdG from the control group, and we observed that the early morning samples show the highest elevation, whereas the 2 h sample did not show any significant difference. These results may suggest that the products of repaired DNA in the form of 8-OHdG are excreted into the urine without any further metabolic processing, and also the presence of the modified nucleoside (8-OHdG) in urine represents the primary repair product of oxidative DNA damage *in vivo*, presumably nucleotide excision repair [44]. Therefore, determination of the level of 8-OHdG directly reflects the oxidative damage of cellular DNA.

Levels of 8-OHdG also depend on certain baseline parameters such as total dose, body weight and age. We observed that the total dose and body weight were significantly associated with the increment of 8-OHdG, which has been previously associated with radiation induced oxidative damage [45], whereas body weight directly relates to the total dose distribution. The positive association of increasing age with the formation of 8-OHdG is plausible because oxidative stress is known to increase during aging [46]. Recent studies suggest that age-associated functional losses are due to the accumulation of ROS- and nitrogen species-induced damages as per the oxidative stress theory [47].

In this study we observed a weak correlation between mean γH2AX formation and the level of 8-OHdG in urine samples (Fig. 8). A stronger relationship may be possible only when factors such as lifestyle habits (e.g. tobacco smoking), metabolic activity and other types of internal and external stressor and epigenetic factors are considered [48, 49].

In summary, the study observed a significant amount of DNA damage after WB ^18^F-FDG PET/CT, which corroborates previous observations. Additionally, our study showed that the level of 8-OHdG in urine sample also increased with the DNA damage, as an integrated, WB indicator of oxidative stress and DNA repair processes. Larger population studies are needed to confirm these observations, describe the fine-scale timing of changes in the biomarker levels after exposure, and further clarify any potential risks to patients from PET/CT procedures.

## Supplementary Material

Supplementary_Material_rrab025
