# Supplementary material for: An evaluation of DNA double strand break formation and excreted guanine species post whole body PET/CT procedure
Source: J Radiat Res. 2021 May 24;62(4):590–9. doi: 10.1093/jrr/rrab025 (PMC8273794; doi:10.1093/jrr/rrab025)
Supplement: Supplementary_Material_rrab025 [file supplementary_material_rrab025.docx]

**Supplementary Material**

**
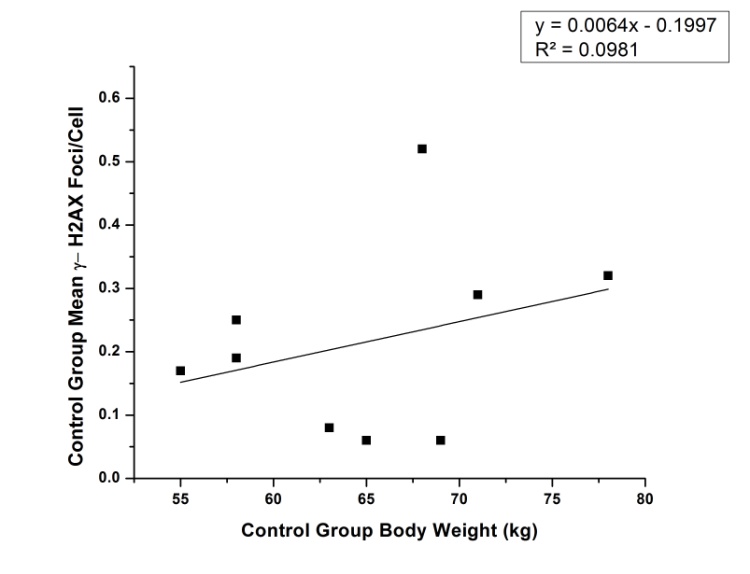
**

**Supplementary figure 1:** Graphical representation of correlation between body weight (kg) and mean γ-H2AX foci/cell of control group


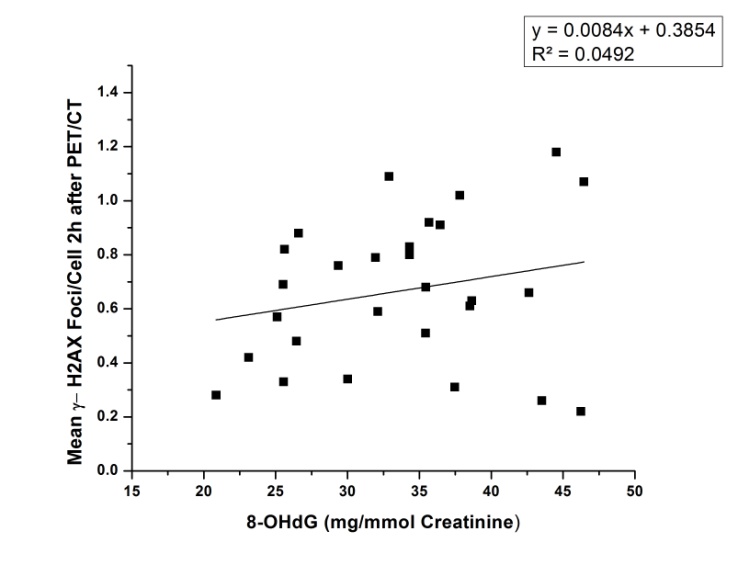


**Supplementary figure 2:** Graphical representation of correlation between mean γ-H2AX foci/cell at 2 h after PET/CT sample and quantified 8-OHdG at 24 h collected sample.


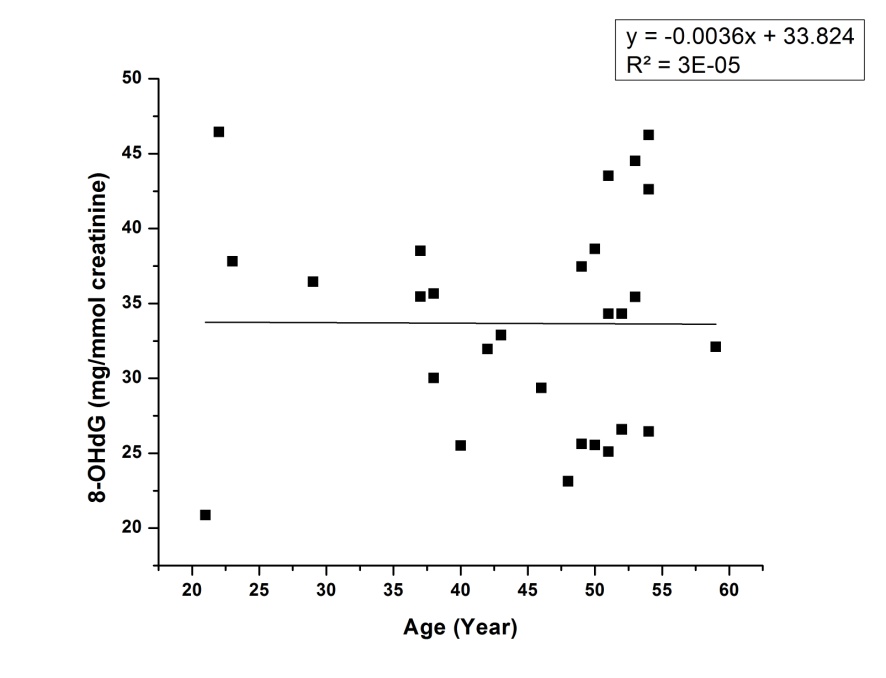


**Supplementary figure 3:** Graphical representation of correlation between quantified 8-OHdG (mg/mmol creatinine) and age of the individual patients.

**Supplementary table 1**

The details of study parameters recorded at individual patients’ level.

| **Age (Year)** | **Sex** | **Weight (kg)** | **Cancer name or type** | **PET (mSv)** | **CT (mSv)** | **Total Received Dose (mSv)** | **Creatinine (mg/dl)** | **CBG (mg/dl)** |
| --- | --- | --- | --- | --- | --- | --- | --- | --- |
|  |  |  |  |  |  |  |  |  |
| 37 | F | 62 | Right-breast cancer | 9.42 | 18.54 | 27.96 | 0.6 | 71 |
| 54 | F | 86 | Left-breast carcinoma | 13.07 | 18.12 | 21.19 | 0.8 | 101 |
| 50 | F | 48 | Ovary & endometrial cancer | 7.31 | 19.82 | 27.13 | 0.5 | 83 |
| 22 | F | 62 | Esophagus cancer | 9.51 | 17.95 | 27.46 | 0.5 | 116 |
| 21 | F | 95 | Squamous cell carcinoma cervix | 14.44 | 20.54 | 34.98 | 0.6 | 84 |
| 23 | F | 50 | Hard palate squamous cell carcinoma | 7.6 | 21 | 28.6 | 0.6 | 105 |
| 50 | F | 66 | Esophagus cancer | 9.86 | 19.61 | 29.47 | 0.6 | 138 |
| 54 | F | 55 | Squamous cell carcinoma cervix | 8.93 | 18.11 | 27.04 | 0.5 | 140 |
| 38 | F | 61 | Left ovary cancer | 9.3 | 18.6 | 27.9 | 0.7 | 100 |
| 40 | F | 69 | Cancer of unknown primary, Liver Lesions and malignant pleural fluid | 10.48 | 19.8 | 30.28 | 1 | 124 |
| 46 | F | 78 | Stomach cancer | 11.85 | 20.54 | 32.39 | 0.6 | 97 |
| 29 | F | 63 | Malignant melanoma (Rectum) | 9.57 | 17.23 | 26.8 | 0.7 | 95 |
| 59 | F | 67 | Left breast invasive ductal carcinoma | 10.18 | 17.85 | 28.03 | 0.9 | 123 |
| 53 | F | 84 | Squamous cell carcinoma cervix | 12.9 | 19.12 | 32.02 | 0.6 | 107 |
| 42 | M | 67 | Renal cell carcinoma | 10.18 | 17.39 | 27.57 | 1 | 90 |
| 49 | M | 53 | Left buccal mucosa carcinoma | 8.05 | 16.12 | 24.17 | 0.6 | 79 |
| 52 | M | 60 | Stomach cancer | 9.12 | 17.11 | 26.23 | 0.7 | 114 |
| 38 | M | 59 | Prostate carcinoma | 8.96 | 19.01 | 27.97 | 0.5 | 99 |
| 52 | M | 40 | Left-lung cancer | 6.08 | 17.01 | 23.09 | 0.9 | 111 |
| 37 | M | 59 | Left-lung non-small-cell lung carcinoma | 8.92 | 17.23 | 26.15 | 1 | 89 |
| 53 | M | 47 | Adeno-carcinoma right lung | 7.14 | 16.98 | 24.12 | 0.7 | 129 |
| 51 | M | 61 | Round cell tumor | 9.17 | 17.19 | 26.36 | 0.8 | 109 |
| 54 | M | 73 | Pyriform sinus carcinoma | 11.87 | 17.45 | 29.32 | 0.9 | 150 |
| 51 | M | 53 | Renal cell carcinoma | 7.44 | 17.58 | 25.02 | 0.8 | 96 |
| 43 | M | 68 | non-Hodgkin lymphoma | 9.98 | 17.1 | 27.08 | 0.7 | 81 |
| 48 | M | 55 | Right-lung mass | 8.15 | 15.89 | 24.04 | 1 | 98 |
| 51 | M | 66 | Transitional cell carcinoma right ureter | 10.03 | 17.89 | 27.92 | 1 | 94 |
| 49 | M | 77 | Renal cell carcinoma | 11.7 | 18.15 | 29.85 | 0.6 | 115 |
